# CDK4/6 Inhibitors in Melanoma: A Comprehensive Review

**DOI:** 10.3390/cells10061334

**Published:** 2021-05-28

**Authors:** Mattia Garutti, Giada Targato, Silvia Buriolla, Lorenza Palmero, Alessandro Marco Minisini, Fabio Puglisi

**Affiliations:** 1 CRO Aviano National Cancer Institute IRCCS, 33081 Aviano, Italy; lorenza.palmero@cro.it (L.P.); fabio.puglisi@cro.it (F.P.); 2 Department of Medicine (DAME), University of Udine, 33100 Udine, Italy; giadatargato@gmail.com (G.T.); silviaburiolla@outlook.it (S.B.); alessandro.minisini@asufc.sanita.fvg.it (A.M.M.)

**Keywords:** CDK4/6, CDK4, CDK6, melanoma, Palbociclib, Ribociclib, Abemaciclib

## Abstract

Historically, metastatic melanoma was considered a highly lethal disease. However, recent advances in drug development have allowed a significative improvement in prognosis. In particular, BRAF/MEK inhibitors and anti-PD1 antibodies have completely revolutionized the management of this disease. Nonetheless, not all patients derive a benefit or a durable benefit from these therapies. To overtake this challenges, new clinically active compounds are being tested in the context of clinical trials. CDK4/6 inhibitors are drugs already available in clinical practice and preliminary evidence showed a promising activity also in melanoma. Herein we review the available literature to depict a comprehensive landscape about CDK4/6 inhibitors in melanoma. We present the molecular and genetic background that might justify the usage of these drugs, the preclinical evidence, the clinical available data, and the most promising ongoing clinical trials.

## 1. Introduction

Historically, metastatic melanoma was considered a highly lethal disease [1]. However, recent advances in drug development have allowed a significative improvement in the prognosis of patients with melanoma. In particular, BRAF/MEK inhibitors and anti-PD1 antibodies have entirely revolutionized the management of this disease [2]. Despite their relevant efficacies, several unmet clinical needs still exist. For example, the response rate of anti-PD1 therapy, which is nearly 40%, is still unsatisfactory [2,3]; on the other hand, BRAF/MEK inhibitors are often unable to control the disease for very long periods because of the development of resistance [4,5]. To overtake this challenges, new clinically active compounds are highly needed. 

CDK4 pathway is a frequently altered signaling in melanomas [6]. Considering the current clinical availability of CDK4/6 inhibitors, increasing interest has emerged in CDK4/6 inhibition in melanoma. Indeed, several clinical trials are testing the efficacy of these compounds, especially as part of a multidrug regimen. 

Herein we review the available literature to depict a comprehensive landscape about CDK4/6 inhibitors in melanoma. We present the molecular and genetic background that might justify using these drugs, the preclinical evidence, the clinical available data, and the most promising ongoing clinical trials.

## 2. CDK4/6 Pathway

The cell division cycle (CDC) is the result of the dynamic balance between pro-mitotic and pro-apoptotic/arrest signals, which interplay in a regulatory network [7,8].

Cell progression through the CDC phases requires overcoming critical points, such as the restriction point (R), the gatekeeper of the G1-S transition, and the cell checkpoints [9]. These transitions are mainly driven by the sequential activation of distinct heterodimeric complexes, each constituted by the association of a cyclin protein and a cyclin-dependent kinase (CDK), with regulatory and catalytic functions, respectively [10].

**Figure 1:**
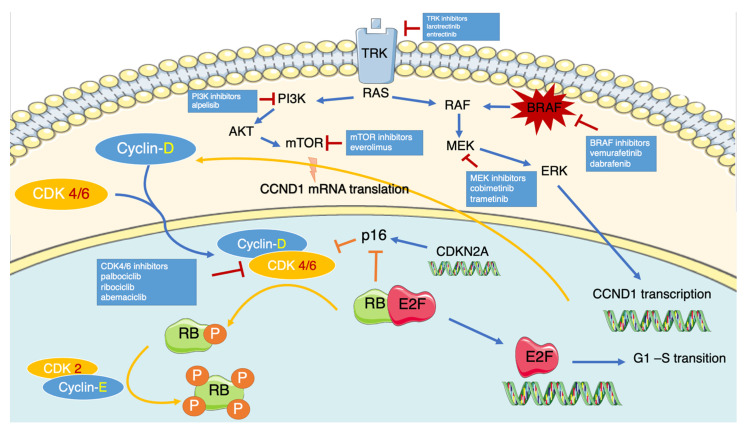
Graphic representation of the most relevant pathways, and their interconnection, that regulate the p16ink4A-cyclin D-CDK4/6-RB pathway. The stimulation of tyrosine kinase receptors on the cell membrane by mitogen stimuli activates the Ras axis, leading to the transcription of CCND1 gene with an increased expression of Cyclin-D. In parallel, the PI3K pathway (PI3K-Akt-mTor) regulates the downstream translation of CCND1 mRNA via mTor. In cellular cytosol, the Cyclin D1 forms complexes with its binding partners, CDK4/6, and translocates to the nucleus. Here, mutually with the downstream complex CyclinE-CDK2, it hyperphosphorylates the RB protein, thus inactivating it. Subsequently, E2F is released and drives the transcription of the genes involved in the G1-S transition. This pathway is negatively regulated by some natural inhibitors, such as p16INK4A, that inhibit the assembly of the Cyclin D-CDK4/6 complex. Moreover, p16INK4A is usually suppressed by the hypophosphorylated form of RB. The CDK4/6 mediated-hyperphosphorylation of RB causes its inactivation and relieves the RB1-mediated suppression of p16INK4A.

Every CDC phase is specifically regulated by a cyclin/CDK complex, and the progression from G1 to S phase is triggered by the cyclin-D-CDK4/6 interaction (Figure 1) [11].

Cyclin D protein, encoded by the CCDN1 gene, is involved in several intracellular pathways, both dependent and independent on CDK4/6, such as promoting cellular proliferation and modulation of mitochondrial activities, DNA damage repair, and cell migration [12]. 

During the CDC the levels and the sub-cellular distribution of cyclin D change with an oscillatory behavior [13], in response to proliferative and mitogenic stimuli; following the binding of growth factors to their tyrosine kinase receptors (e.g., IGFR, c-kit, FGFR), the activated RAS/RAF/MEK/ERK cascade induces the transcription of CCDN1 and consequently the increase in cyclin D expression in the early G1 phase [9,12]. After the heterodimerization of cyclin D with CDK4/6, the activated complex moves to the nucleus and, co-operating with cyclin-E/CDK2, induces the hyperphosphorylation and the consequent impairment of three proteins, all with growth suppressive properties: the tumor suppressor Retinoblastoma protein (RB), the retinoblastoma-like protein 1 (p107) and the retinoblastoma-like protein 2 (p130) [14]. As a result, the E2F transcription factor dissociates from RB and can exert its activity, priming the transcription of genes required for DNA replication and promoting the progression of the cell cycle to the S phase.

To avoid an aberrant cellular proliferation, this process is inhibited by the INK4 (including p16^ink4A^, p15^ink4B^, p18^ink4C^, p19^ink4D^), the Cip, and the Kip (p21^CIP1^ and p27^KIP1^) family proteins [15]. The INK4 proteins specifically bind to the CDK4/6 units and prevent the assembly of the cyclin-D-CDK4/6 complex while p21^CIP1^ and p27^KIP1^ interact both with the cyclins and the CDKs units, stabilizing them. As a result of this inhibition, the cell stops at the G1-S transition phase.

Additional inhibitory control is provided by the negative feedback loop promoted by the interaction between p16^ink4A^ and RB. Physiologically, p16^ink4A^ is suppressed by the activated (hypophosphorylated) form of RB; accordingly, the hyperphosphorylation of RB mediated by CDK4/6 relieves p16^ink4A^ from the suppression. 

In addition to the RB-dependent mechanism (defined as *canonical pathway*), the complex cyclin-D-CDK4/6 directly regulates the cell-cycle progression through the modulation of FOXM1, MYC, and SMAD3 expression respectively involved in senescence, apoptosis, and G1-S transition.

## 3. CDK4/6 Pathway Dysregulation in Melanoma

Dysregulation of the p16^ink4A^-cyclin D-CDK4/6-RB pathway is common in many cancer types [16] and is recognized in up to 90% of melanomas, with alterations occurring in many nodes of this axis [6,17,18]. 

These changes include loss or inactivation of CDKN2A (encoding for p16), aberration or overexpression of CCND1, and mutation or amplification of CDK4/6 [19,20]. These aberrations are responsible for the downregulation of the inhibitory control system or the hyperactivation of the pro-mitotic signaling pathways. 

CDKN2A alterations, comprehensive of deletion, mutation, or promoter methylation, lead to loss of expression of p16 with lack of control on CDK4/6-mediated cell proliferation. A higher prevalence of these modifications has been observed in melanoma tissues compared to benign nevi [21], suggesting their pivotal role in melanoma genesis. Moreover, germline CDKN2A mutations have been shown to increase the susceptibility to develop melanomas [22,23,24]. 

CDK4 mutations in arginine residue 24 (CDK4^R24C^) are currently reported in melanoma patients with a familial predisposition and prevent the binding of p16 to the catalytic subunit, triggering constitutive activation of the kinase [22], and conferring a biological behavior similar to p16 loss, as proved by pre-clinical models [25,26]. 

A critical role in the oncogenic evolution of the p16^ink4A^-cyclin D-CDK4/6-RB pathway is played by CCND1, a node where many upstream stimuli converge [27]. 

The prevalence of the amplification of CCND1 is controversial [28,29] and the frequency differs among melanoma types, with an established higher detection rate in acral melanoma [30,31,32]. Gene copy numbers and amplification levels are frequently associated with the expression of cyclin D, but, in contrast, the overexpression of the cyclin D does not necessarily follow gene expression alterations, suggesting the influx of other pathways [29]. Cyclin D may be upregulated in response to BRAF and PI3K mutations, which lead to the increase in CCND1 gene transcription and mRNA translation, respectively [33]. 

Intriguingly, as shown in 2005 by Curtin et al. [34], different types of melanomas present distinct aberrations [35], suggesting a strong link between the mechanism of melanoma development/progression, biological behavior, and response to target therapy.

Additionally, ultraviolet radiations doubly intertwine with the p16^ink4A^-cyclin D-CDK4/6-RB pathway. Pre-clinical evidence showed that UV light could induce the p16^ink4A^ expression for the UV-lesions repairment [36,37] and both enhance melanomagenesis by promoting p16 loss or CDK6 amplification [38,39].

## 4. Pharmacology of CDK4/6 Inhibitors

Palbociclib, Ribociclib, and Abemaciclib are the third-generation of orally administered CDK4/6 inhibitors. Through a competitive and reversible bound to the ATP pocket of the inactive kinase, that strongly inhibits both CDK4 and CDK6 preventing the RB hyperphosphorylation and thus causing cell cycle arrest (Table 1) [40]. These drugs show little or absent suppression of other CDK family members: this results in a reduction of adverse events associated with broad-spectrum CDK blockade, as observed with first- and second-generation CDK inhibitors [41]. 

While Palbociclib inhibition activity is similar for CDK4 and CDK6, Ribociclib and Abemaciclib are more selective on CDK4 than CDK6. Abemaciclib showed higher inhibitor potency among these drugs, especially on CDK4 [40,45]. Furthermore, it has shown in vitro activity against other cyclin-dependent kinases as CDK1, CDK2, CDK7, and particularly CDK9, but also against other pathways involved in cellular proliferation and survivorship [46]. These differences in selectivity and CDK inhibition ratio may be responsible for a different safety profile and dose schedules, assuming clinical relevance [19]. These three agents are associated with hematological and gastrointestinal adverse events because of cell cycle inhibition in highly proliferative tissues, like bone marrow and gastrointestinal mucosa. In particular, targeting CDK6 has been associated with alterations in hematopoiesis due to a cytostatic effect [19]. Therefore, while Palbociclib and Ribociclib are frequently responsible for leukopenia, neutropenia, and anemia, Abemaciclib, has higher selectivity for CDK4, results in less hematological toxicity and is mainly related to gastrointestinal disorders, like nausea and diarrhea, potentially mediated by CDK9 inhibition [47]. Other concerning side effects are an increased risk of thromboembolism and Ribociclib has been associated with prolonged QT interval and hepatotoxicity [48,49].

These three different CDK4/6 inhibitors show a similar pharmacokinetic profile. However, after oral administration, Ribociclib is more rapidly absorbed compared to Abemaciclib and Palbociclib, the last showing a longer maximum concentration time (T_max_) [50,51]. Palbociclib absorption is influenced by food intake and gastric pH, with lower drug concentration levels observed in patients assuming Palbociclib in fasting condition or in association with proton pump inhibitors [45]. On the contrary, Ribociclib and Abemaciclib absorption are not significantly influenced by food intake, though high fat and caloric meals slightly increase Abemaciclib plasma concentration [52]. Compared to other CDK 4/6 inhibitors, Abemaciclib shows a shorter mean half-life (T_1/2_), and thus more frequent dosing is required to maintain stable plasma levels capable of sustaining cell cycle arrest [53].

Palbociclib and Ribociclib present a higher distribution volume due to their moderate binding to human plasma proteins [45]. At the same time, Abemaciclib shows greater lipophilicity and penetrates more efficiently through the blood-brain barrier, as observed in some clinical and preclinical studies [53,54]. In addition, Palbociclib and Ribociclib are both substrates of breast cancer resistance protein (BCRP; ABCG2) and P-glycoprotein (P-gp; ABCB1), two efflux transporter proteins responsible for cerebrospinal fluid drugs removal [54].

These drugs undergo hepatic metabolism primarily mediated by CYP3A4. Thus, concomitant use of potent CYP3A4 inhibitors or the presence of specific genetic polymorphisms can lead to higher CDK4/6 inhibitors blood levels with increased risk of toxicity with clinical practice impact [54]. 

## 5. Preclinical Activity of CDK4/6 Inhibitors in Melanoma

### 5.1. CDK4/6 Inhibitors as Single Agents

Since the CDK4/6 pathway is frequently dysregulated in melanoma, it could be anticipated that CDK4/6 inhibitors have a high degree of activity against this disease. Counterintuitively, clinical data have documented a limited role of these agents when used alone, suggesting an intrinsic resistance of melanoma to CDK4/6 inhibitors (see below). Although no definitive conclusions exist regarding the exact resistance mechanisms, some preclinical studies have shed light on this topic. 

#### Resistance Mechanisms to CDK4/6 Inhibitors

Several mechanisms of resistance to CDK4/6 inhibitors have been suggested. One putative mechanism of resistance resides in the PI3K-AKT-mTOR signaling cascade. For example, in the elegant study of Hayes et al., through a gene-expression- and a gene-silencing-system, it was shown that receptor tyrosine kinases (RTK)-PI3K-AKT-mTOR pathway activity impairs sensitivity of melanoma cells to Palbociclib [55]. Similarly, Yoshida et al. demonstrated an increased mTOR signaling drives resistance to CDK4/6 inhibitors [56]. Of note, both the loss of RB and the overexpression of FXR1 seem to cause an increase of SLC36A1 levels, which, in turn, promote mTOR signaling.

Another putative mechanism of resistance stems from the reduced activity of p21 [57]. In detail, upon treatment with CDK4/6 inhibitors, melanoma cells increase the levels of cyclin D1, which is a master regulator of cell cycle progression. Cyclin D1, in turn, sequesters p21 and other CDK-inhibitors (e.g., p27). Since p53 transcriptionally regulates p21, Vilgem et al. tried to increase p21 levels through inhibition of MDM2, which is a p53 degrader. Indeed, the coadministration of MDM2 inhibitors and CDK4/6 inhibitors leads to tumor regression [57]. The antagonistic role of MDM2 against CDK4/6 inhibitors was also confirmed by others [58]. Similarly, also the p53-degraders MDM4 has shown an antagonistic role against CDK4/6 inhibitors [59].

Collectively, these data suggest that combining mTOR inhibitors and/or MDM2/MDM4 inhibitors might overcome the intrinsic resistance to CDK4/6 inhibitors in melanoma. All these drugs are already clinically available. In addition, data from other cancer types suggest several more putative mechanisms of resistance [60]: CDK4/6 overexpression [61], p16 amplification [62,63], upregulation of FGFR pathway [64,65], alteration of Hippo pathway [66], activation of CDK2 [67,68], autophagy [69], and epigenetic alterations [70,71].

### 5.2. CDK4/6 Inhibitors Combined with Immunotherapy

Immunotherapy has deeply changed the way to treat melanoma. Indeed, it could achieve very long-term disease control or even disease remission [2]. However, only a fraction (35–60%) of patients respond to this therapy, and nearly 40% of them experienced resistance to immunotherapy within three years [2,3]. There is a mounting interest in compounds that enhance efficacy or that reduce resistance to immunotherapy. CDK4/6 inhibitors are a promising drug class that showed an interesting synergistic activity with immunotherapy. 

Zhang et al. [72] showed that CDK4/6 inhibition could stimulate PD-L1 expression, which, in turn, impairs antitumor immunity and reduces tumor-infiltrating lymphocytes. However, the coadministration of a CDK4/6 inhibitor with anti-PD-1 immunotherapy enhanced tumor regression and increased overall survival in mouse cancer models. Similarly, Yu et al. [73] reported that CDK4 pathway aberrations confer an intrinsic resistance to anti-PD-1 immunotherapy, and these results were validated in a cohort of 85 patients with melanoma. In addition, they showed an enhanced efficacy from the combination of an anti-PD-1 antibody with Palbociclib compared to monotherapies. Coherently, Jerby-Arnon et al. demonstrated in an elegant way the pro-immunogenic potential of CDK4/6 inhibitors. In particular, through the single-cell RNA sequencing from 33 melanomas, it was shown that cold neoplastic niches were characterized by a resistant transcriptomic profile linked with T-cell exclusion and immune evasion. This program, which predicts reduced clinical response to immunotherapy, could be reverted by CDK4/6 inhibition [74]. 

From a mechanistic point of view, several putative pro-inflammatory activities of CDK4/6 inhibitors have been described. Among them, three could be particularly relevant in melanoma. First, CDK4/6 inhibitors have been shown to increase Type III interferon production and cancer antigen presentation [75,76]. Second, CDK4/6 inhibitors could suppress regulatory T-cell proliferation, indirectly enhancing anticancer immune surveillance [75]. Third, CDK4/6 inhibitors significantly increase T-cell activation and functions, directly enhancing anticancer immune functions [77]. 

Comprehensively, preclinical data suggest a promising synergistic potential from CDK4/6 inhibitors and immunotherapy. Confirmatory clinical trials are ongoing (see below).

### 5.3. CDK4/6 Inhibitors Combined with BRAF/MEK Inhibitors

Nearly 50% of melanomas have a mutation in the BRAF gene [78], and BRAF inhibitors have shown tremendous efficacy in this subgroup of patients [79]. However, resistance to BRAF inhibition arises quickly [79]. In order to delay cancer progression, the combination of BRAF- and MEK-inhibitors has been shown to increase response rate and duration of response [4,5]. Nevertheless, the development of acquired resistance to this therapy is a common problem that often stems from the reactivation of the MAPK pathway, which could stimulate CDK4/6 signaling [80]. Indeed, preclinical evidence supports the role of combined inhibition of RAF/MEK and CDK4/6 [81,82]. From a molecular point of view, several mechanisms of synergism from combined BRAF/MEK- and CDK4/6-inhibition have been proposed. 

Yoshida et al. showed that prolonged inhibition of CDK4/6 signaling could overcome the resistance to BRAF-inhibitors through the induction of a state of senescence [83]. Since senescence was previously linked to increased cancer clearance by the immune system [84], Teh et al. have evaluated a possible immune-stimulating effect of combined CDK4/6-MEK-inhibitors. Indeed, they found that the combination increases tumor immunogenicity, intra-tumoral CD8 T-cell recruitment, and the expression of CD137L, a T-cell costimulatory molecule on immune cells [85].

Another mechanism that could be exploited by the combination of CDK4/6- with BRAF/MEK-inhibitors is the RB/cyclin D1 axis. In particular, Abemaciclib, a CDK4/6 inhibitor, can impair cell cycle progression through partial inhibition of RB phosphorylation. However, as a compensatory effect, there is an increase in cyclin D1 levels. Combined treatment with LY3009120 (a pan-RAF inhibitor) with Abemaciclib results in complete inhibition of RB phosphorylation and a reduction in cyclin D1 levels translating into a significant cell cycle arrest [78]. The increased BRAF degradation caused by CDK4 inhibition could, at least in part, explain the synergistic effect of combined therapies [86]. Of note, it appears that upfront triple therapy (CDK4/6-BRAF-MEK-inhibitors) may have superior efficacy compared to the addition of CDK4/6 inhibitor after tumor acquisitions of BRAF/MEK inhibitors resistance [87].

Notably, it seems that NRAS-mutant melanoma could be particularly sensitive to the combined CDK4/6/BRAF/MEK inhibition [55]. For example, in the elegant work of Kwong et al., it was shown that in NRAS-mutant melanomas, pharmacological inhibition of MEK could drive to apoptosis but not cell-cycle arrest, as it would happen after the abrogation of NRAS activity. However, the combined inhibition of MEK and CDK4 supersedes this difference leading to a substantial increase in treatment efficacy [88].

Finally, some preliminary evidence suggests that increased mTOR signaling could confer resistance to CDK4/6/BRAF/MEK triple therapy [89]. 

Comprehensively, the combined inhibition of CDK4/6- and BRAF/MEK-axis has shown preclinical encouraging results. Confirmatory clinical trials are ongoing (see below). 

## 6. Clinical Activity of CDK4/6 Inhibitors in Melanoma

Multiple clinical trials evaluated CDK4/6 inhibitors as single agent regimen or in combination with BRAF ± MEK inhibitors as first or subsequent line of therapy in patients with advanced solid tumors, including melanoma (Table 2).

### 6.1. CDK4/6 Inhibitors as Single Agents

Patnaik and colleagues tested Abemaciclib in a Phase I study that enrolled patients affected by different cancer types, like breast cancer, non-small cell lung cancer, glioblastoma, melanoma, and colorectal cancer. They evaluated the safety, the pharmacokinetic, and pharmacodynamic profiles and the anti-tumor activity of Abemaciclib in 225 patients. Among them, 26 had a diagnosis of advanced melanoma: one patient achieved RECIST partial response (PR), while six patients had stable disease (SD), for an overall disease control rate of 27%. The patient with metastatic melanoma that achieved a PR had a tumor with molecular alterations (NRAS mutation and copy-number loss at the INK4 locus) that induced aberrant CDK4 and CDK6 activation [53]. Abemaciclib was subsequently studied by Sahebjam et al. in patients with brain metastases from non-small cell lung cancer and melanoma. They demonstrated limited intracranial activity in this population. In fact, no confirmed intracranial response was observed (objective intracranial response rate of 0% for both cohorts). Based on the efficacy results, the authors concluded that no further studies are warranted for Abemaciclib monotherapy in this patient population [92].

The therapeutic benefit of Palbociclib has been observed in two Phase 1 trials conducted in Rb-positive solid tumors and non–Hodgkin lymphomas. These studies involved 33 and 41 patients, respectively, including four and six that were diagnosed with melanoma. Overall, none of the melanoma patients achieved a PR, while SD was recorded in one patient with melanoma in both studies [90,93]. 

The anti-tumor activity of Palbociclib was also tested in a trial that demonstrated preliminary activity in patients with acral lentiginous melanoma and gene aberrations in the CDK pathway [94]. Tang et al. described two case reports of melanoma patients with copy number variations of CDK4 pathway-related genes who received Palbociclib. Although no complete or partial responses were observed in these patients, the diseases were controlled for over six months after the failure of chemotherapy or immunotherapy, indicating that the CDK4 pathway is a potential therapeutic target [95].

In another Phase I dose-escalation study, Ribociclib used as a single agent was administered in patients with wild-type RB advanced solid tumors or lymphomas, with the aim to assess the safety, pharmacokinetics, pharmacodynamics, and preliminary activity. Overall, 132 patients were enrolled: more than half had previously undergone radiation therapy, and nearly three-quarters had received two or more prior systemic therapies. Three were diagnosed with melanoma, and one achieved a PR [50].

### 6.2. CDK4/6 Combined with Other Agents

The efficacy of CDK4/6 inhibitors in association with target therapy has been tested in several trials, mostly Phase I or II, with interesting results.

Ribociclib was evaluated with Binimetinib (NRAS/MEK inhibitor) [50] or Encorafenib (BRAF inhibitor) [96] in two different Phase I/II clinical trials in advanced NRAS and BRAF mutant melanoma. Preliminary results from Ribociclib and Binimetinib combination were presented in the abstract form at ASCO 2014 by Sosman et al. 14 pretreated patients were enrolled and Ribociclib was administered once daily for 21 days while Binimetinib was administered twice daily continuously. This combination showed an interesting antitumor activity and safety, in fact six patients achieved PR (43%) and six had SD. Several patients experienced early tumor shrinkage with major symptomatic improvement. The most common treatment-related toxicities were phosphokinase elevation, creatinine elevation, acneiform rashes, nausea, edema, leukopenia, and neutropenia [97]. Ribociclib and Encorafenib, in turn, demonstrated clinical activity and acceptable tolerability profile in patients naïve or pretreated (median three prior regimens). Ribociclib was administered with a three week on and one week off schedule, while Encorafenib was given continuously. Of nine patients evaluable for response, two had PR and five had SD and, of these, three were BRAF inhibitors-naïve and three BRAF inhibitors-pretreated. Principal adverse events registered were palmar-plantar hyperkeratosis, flushing, pruritus, rash, alopecia, dry skin, dysgeusia, fatigue, myalgia and nausea [96]. 

Preliminary results from a Phase IB/II dose-escalation study evaluating triple combination therapy with BRAF and MEK inhibitors (Encorafenib plus Binimetinib associated with Ribociclib) were presented as abstract by Ascierto et al. at ASCO 2017. The trial enrolled patients with BRAF-V600-mutant solid tumors. The triple combination was tested in BRAF-V600 melanoma patients naïve to prior BRAF inhibitor treatment. More than half of patients (52.4%) showed a reduction in tumor size, including four complete responses (CR), 18 PR, and 15 SD. Median PFS was nine months. No dose-limiting toxicities have been reported. The most common adverse events were neutropenia, alanine transaminase elevations, diarrhea, and anemia [98].

Finally, Palbociclib and Vemurafenib were tested in an open-label Phase I-II trial involving patients with stage IIIC or IV BRAF-V600^E/K^ mutant metastatic melanoma harboring CDKN2A loss and wild type-RB1-expression. This study aimed to establish the maximum tolerated dose of Palbocliclib associated with Vemurafenib. The schedule for Palbociclib was 14 days-on and seven-off, while Vemurafenib was taken continuously twice daily. Secondary endpoints included the best response, OS, and PFS. Patients have been stratified into two groups accordingly to previous BRAF inhibitor treatment (Group 1: no vs. Group 2: yes). The overall response rate of BRAF inhibitor pretreated patients was 25% and SD was registered in 50%. Intriguingly, the median PFS and OS of Group 2 were 9.3 and 13.2 months, respectively. Therefore, the authors concluded that significant clinical benefit was achieved in heavily pretreated melanoma patients [99]. 

Regarding immunotherapy, clinical trial that evaluating combinations of CDK4/6 inhibitors and immunotherapy in advanced solid tumors, including melanomas, are ongoing. As previously reported, there are some preclinical evidence for synergy. 

These data will be useful to broaden the therapeutic horizon in patients with advanced melanoma. 

## 7. Ongoing Clinical Trials with CDK4/6 Inhibitors in Melanoma

To outline the current trends of ongoing clinical trials in melanoma with CDK 4/6 inhibitors, we performed a keywords-driven search (compound, molecular and commercial names of Palbociclib, Ribociclib, and Abemaciclib respectively, restricted for melanoma malignancy) on clinicaltrials.gov and manually annotated the trials of interest. We included only clinical trials conducted in a metastatic and advanced setting not amenable to radical locoregional treatment, including both cutaneous and mucosal variants and regardless of BRAF and NRAS mutational status. The CDK 4/6 inhibitors administration could have been scheduled as a first or subsequent line of treatment, both in a single agent- and in combination-therapy.

Although nine clinical trials were identified, six among them are conducted specifically in melanoma disease and therefore were considered of greater interest: four were Phase II and one was Phase I, while one trial was a Phase I/II study. Thus, the planned number of enrolled patients was from low to intermediate. Objective response rate (ORR) is the primary endpoint of the great part of Phase II studies (three out four), while Phase I trials focus on safety and dose-limiting toxicities (DLTs) of investigated drugs (Table 3). 

Two studies are designed to evaluate the DLTs and the ORR (NCT02974725 and NCT04417621, respectively) during combination therapy with Ribociclib and LXH254, a BRAF and CRAF inhibitor with antitumor activity in MAPK-driven tumor models [100]. The first trial is currently enrolling both NRAS mutant cutaneous melanomas and KRAS or BRAF mutant non-small cell lung cancers progressed after standard of care therapy, while the second one is focused on pre-treated BRAF or NRAS mutant melanomas.

The PLATforM study (NCT03484923) is, to the best of our knowledge, the only ongoing clinical trial with a CDK 4/6 inhibitor combined with an anti-PD-1 drug focused on melanoma disease. In this study, patients with previously treated unresectable or metastatic melanoma, both mutant and wild type BRAF, are randomized to receive Spartalizumab combined with a second agent between Ribociclib, Capmatinib, Canakinumab, and LAG525. 

The CELEBRATE (NCT04720768) and the LOGIC-2 (NCT02159066) trials are intriguingly evaluating a three-drug regimen with Encorafenib and Binimetinib plus a CDK 4/6 inhibitor in BRAF V600 positive patients. The CELEBRATE study admits both naïve and previously treated patients with BRAF and MEK inhibitor combination therapy, but also with checkpoint inhibitors or chemotherapy. In the LOGIC-2 trial, patients who progressed to first-line Encorafenib and Benimetinib continue the combination therapy and are assigned to add a third drug (including Ribociclib) according to the melanoma genetic profile. 

A mutation-driven approach is also proposed in the MATCH (NCT02465060) and in the MatchMel (NCT02645149) studies. The second one focused on melanoma patients. In this trial, BRAF and NRAS wild-type patients who progressed to standard therapy are subjected to comprehensive gene testing and afterward assigned to a targeted therapy matched for their genetic result. Treatment with Ribociclib plus Trametinib has been planned for patients with CCND1, CDK4/6, CDKN2A or RAS mutations. 

As observed in other malignancies, in the precision medicine era, there is an increasing tendency to personalize the oncological treatment according to cancer genetic profile and, thus, as reported above, different studies also in melanoma setting are moving in this direction. 

Of note, just one trial comprises a small cohort of treatment naïve patients, while the current trend is to investigate CDK 4/6 inhibitors in association with other drugs after standard first-line treatment failure. Finally, the results of the study that combine CDK4/6 inhibitor with novel agents as LXH254 and LY3214996 (an ERK 1/2 inhibitor) are much awaited [91,100]. 

## 8. Conclusions

Despite noteworthy improvement in melanoma treatment, there is an urgent need for new and effective compounds. CDK4/6 inhibitors have already revolutionized the management of other cancers, notably breast cancer. Although their clinical utility in melanoma is probable, more studies are needed to confirm their relevance in order to include these drugs in clinical practice. However, from the currently available evidence, CDK4/6 inhibitors’ monotherapy might offer a modest benefit at best. On the contrary, adding CDK4/6 inhibitors to BRAF/MEK inhibitors or anti-PD1 therapy might offer a substantial benefit. It will be of particular importance the evaluation of toxicities of the combined regimens and the finding of reliable predictive biomarkers of CDK4/6 inhibitor-efficacy.

## Figures and Tables

**Table 1:** Pharmacologic features of Palbociclib, Ribociclib, and Abemaciclib.

	Palbociclib (IBRANCE^®^) [42]	Ribociclib (KISQUALI^®^) [43]	Abemaciclib (VERZENIOS^®^) [44]
Chemistry	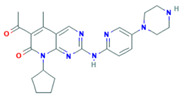	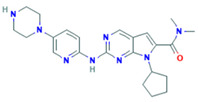	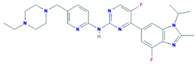
Dose and schedule	125 mg daily three weeks on/one week off	600 mg daily three weeks on/one week off	150 mg twice daily continuously
Administration	Oral	Oral	Oral
Form	Capsule	Tablet	Tablet
Activity IC_50_ CDK4 IC_50_ CDK6 IC_50_ CDK9	CDK4 and CDK6 CDK4 ≈ CDK6 11 nM 16 nM NR	CDK4 and CDK6 CDK4 > CDK6 10 nM 39 nM NR	CDK4, CDK6, CDK1, CDK2, CDK7 and CDK9 CDK4 >> CDK6 2 nM 10 nM 57 nM
PK T_max_ (h) T_1/2_ (h) Vd (L)	4–12 24–34 2583	1–4 30–55 1090	8 17–38 690.3
Lipophilicity (cLog P)	2.7	2.3	5.5
Protein binding	85%	70%	96–98%
Bioavailability	46%	NR	45%
Metabolism	Hepatic (CYP3A4 and SULT2A1)	Hepatic (CYP3A4)	Hepatic (CYP3A4)
Metabolites	Glucuronide conjugate: 1.5%	M13 (CCI284, N-hydroxylation): 22% M4 (LEQ803, N-demethylation): 20% M1 (secondary glucuronide): 18%	M2 (N-desethylabemaciclib): 25% M18 (hydroxy-N-desethylabemaciclib): 13% M20 (hydroxyabemaciclib): 26%
Excretion	Feces: 74% Urine: 17%	Feces: 69.1% Urine: 22.6%	Feces: 81% Urine: 3.4%

**Table 2:** Clinical evidence of CDK4/k inhibitors in melanoma.

Drug	Evidence	N° Patients	NCT Number
Abemaciclib	Phase I dose escalation and tumor-specific cohort study. Primary objective: safety and tolerability. Secondary objectives: pharmacokinetics, evaluate biomarkers, antitumor activity, and establish a recommended dose range	225 total (26 melanoma)	NCT01394016
Abemaciclib	Phase 2, non-randomized study. Primary end point: objective intracranial response rate. Secondary end point: intracranial clinical benefit, PFS, OS and safety.	51 total (23 melanoma)	NCT02308020
Palbociclib	Phase I, dose-finding, non-comparative study. Primary objectives: safety, identifying dose-limiting toxicities (DLT), the maximum administered dose and the maximum tolerated dose (MTD), and to establish the recommended dose for Phase II studies (RP2D). Secondary objectives: characterization of single-dose and steady-state pharmacokinetics (PK) and evaluation of preliminary anti-tumor activity.	33 total (4 melanoma)	[90]
Palbociclib	Phase I, dose-finding, non-comparative study. Primary objectives: safety, DLT, maximum administered dose, MTD. Secondary objectives: characterization of single-dose and PK and evaluation of preliminary anti-tumor activity	41 total (6 melanoma)	NCT00141297
Palbociclib	Phase II, open-labeled study. Primary end point: ORR. Secondary end point: OS, PFS, safety	15 (melanoma)	NCT03454919
Palbociclib	Case report	2 (melanoma)	[91]
Ribociclib	Phase I, dose-escalation study. Primary end point: MTD/recommended dose for expansion (RDE), and DLT. Secondary end point: safety, PK, pharmacodynamics (PD), and preliminary activity of ribociclib	132 total (3 melanoma)	NCT01237236
Ribociclib and Binimetinib	Phase 1b/2 study of LEE011 + binimetinib. Primary objective: estimate MTD/RP2D. Secondary objectives: safety, PK and efficacy.	14 (melanoma)	NCT01781572
Ribociclib and Encorafenib	Phase 1b/2. Primary objective safety and efficacy	18 (melanoma)	NCT01777776
Ribociclib, Encorafenib and Binimetinib	Phase 1b, multicenter study, primary objective: MTD, DLT and ORR	63 (melanoma)	NCT01543698
Palbociclib and Vemurafenib	Phase I–II, multicenter study. Primary objective DLT, secondary objective: efficacy, tolerance and one year survival rate	99 (melanoma)	NCT02202200

**Table 3:** Ongoing clinical trial with CDK4/6 inhibitors in melanoma. DLTs = dose listing toxicities; * melanoma and BRAF and KRAS mutant non-small cell lung cancers.

Study	Phase	Setting	Investigated Drug	N.	Primary Outcome	Current Status	Estimated End
Melanoma specific studies							
NCT04720768 (CELEBRATE)	Ib/II	Metastatic or unresectable untreated or previously treated melanoma BRAF V600 mutant	Encorafenib + Binimetinib + Palbociclib	78	DLTs	Recruiting	December 2023
NCT02974725 *	Ib	Metastatic or advanced cutaneous previously treated melanoma NRAS mutant	LXH254 + Ribociclib	331	DLTs and safety	Recruiting	May 2022
NCT02159066 (LOGIC-2)	II	Metastatic or unresectable melanoma BRAF V600 mutant progressed to prior Encorafenib + Binimetinib	Encorafenib + Binimetinib + Ribociclib	160	ORR	Active, not recruiting	January 2022
NCT04417621	II	Metastatic or unresectable previously treated melanoma BRAF V600 or NRAS mutant	LXH254 + Ribociclib	320	ORR	Recruiting	April 2023
NCT03484923 (PLATforM)	II	Metastatic or unresectable previously treated melanoma	Spartalizumab + Ribociclib	195	ORR	Recruiting	June 2022
NCT02645149 (MatchMel)	II	Metastatic or unresectable melanoma	Trametinib + Ribociclib	1000	Type and frequency of genetic aberrations in BRAF/NRAS wt metastatic melanoma and proportion of BRAF/NRAS wt receiving target therapy	Not yet recruiting (May 2021)	December 2028
Non-melanoma specific studies							
NCT02465060 (MATCH)	II	Metastatic or recurrent previously treated melanoma	Palbociclib	6452	ORR	Recruiting	June 2022
NCT02857270	I	Metastatic melanoma NRAS or BRAF mutant progressed to target therapy	LY3214996 + Abemaciclib	245	LY3214996 DLTs	Recruiting (not yet recruiting for melanoma cohort)	September 2021
NCT02791334 (PACT)	I	Metastatic cutaneous melanoma	LY3300054 + Abemaciclib	215	LY3300054 DLTs	Active, not recruiting	December 2021

## Abbreviations

CDC = cell division cycle. CDK = cyclin-dependent kinase. DLT = dose limiting toxicity. ORR = objective response rate. OS = overall survival. PFS = progression-free survival. RTK = receptor tyrosine kinases.
